# Self-reported outcomes on oral health and oral health-related quality of life in long-term childhood cancer survivors—A DCCSS-LATER 2 Study

**DOI:** 10.1007/s00520-023-07797-1

**Published:** 2023-05-19

**Authors:** Juliette Stolze, Judith E. Raber-Durlacher, Jacqueline J. Loonen, Jop C. Teepen, Cécile M. Ronckers, Wim J. E. Tissing, Andrica C. H. de Vries, Sebastian J. C. M. M. Neggers, Eline Dulmen-den Broeder, Marry M. Heuvel-Eibrink, Helena J. H. van der Pal, A. Birgitta Versluys, Margriet Heiden-van der Loo, Marloes Louwerens, Leontien C. M. Kremer, Dorine Bresters, Henk S. Brand, and on behalf of the DCCSS-LATER Study Group, Martha Grootenhuis, Flora van Leeuwen, Lideke van der Steeg, Geert Janssens, Hanneke van Santen, Margreet Veening, Jaap den Hartoghg, Saskia Pluijm, Lilian Batenburg, Hanneke de Ridder, Nynke Hollema, Lennart Teunissen, Anke Schellekens

**Affiliations:** 1 Princess Máxima Center for Pediatric Oncology, 3584 CS Utrecht, The Netherlands; 2 Department of Oral Biochemistry, Academic Center for Dentistry Amsterdam (ACTA), 1081 LA Amsterdam, The Netherlands; 3 Department of Oral Medicine, Academic Center for Dentistry Amsterdam (ACTA), 1081 LA Amsterdam, The Netherlands; 4 Department of Oral and Maxillofacial Surgery, Amsterdam University Medical Center (UMC), Location AMC, 1105 AZ Amsterdam, The Netherlands; 5 Radboud University Medical Center, 6525 GA Nijmegen, The Netherlands; 6 Division of Childhood Cancer Epidemiology (EpiKiK), Institute of Medical Biostatistics, Epidemiology and Informatics, University Medical Center of the Johannes Gutenberg University Mainz, Mainz, Germany; 7 Department of Pediatric Oncology, Beatrix Children’s Clinic, University Medical Center Groningen, 9713 GZ Groningen, The Netherlands; 8 Department of Pediatric Oncology, Sophia Children’s Hospital, Erasmus Medical Center, 3015 GD Rotterdam, The Netherlands; 9 Department of Internal Medicine, Section Endocrinology, Erasmus Medical Center, 3015 GD Rotterdam, The Netherlands; 10 Emma Children’s Hospital, Amsterdam UMC, Location VUmc, 1105 AZ Amsterdam, The Netherlands; 11 Department of Internal Medicine/Endocrinology, Leiden University Medical Center, 2333 ZA Leiden, The Netherlands; 12 Wilhelmina Children’s Hospital, University Medical Center Utrecht, 3584 EA Utrecht, The Netherlands; 13 Emma Children’s Hospital, Amsterdam UMC, Location AMC, 1105 AZ Amsterdam, The Netherlands

**Keywords:** Childhood cancer, Oncology, Late effects, Childhood cancer survivors, Oral health, Oral health–related QoL

## Abstract

**Purpose:**

The present study aimed to determine the prevalence of self-reported oral problems and the oral health–related quality of life (OHRQoL) in childhood cancer survivors (CCS).

**Methods:**

Patient and treatment characteristics of CCS have been collected in a cross-sectional study, part of the multidisciplinary DCCSS-LATER 2 Study. To assess self-reported oral health problems and dental problems, CCS filled out the ‘Toegepast-Natuurwetenschappelijk Onderzoek’ (TNO) oral health questionnaire. OHRQoL was assessed by the Dutch version of the Oral Health Impact Profile-14 (OHIP-14). Prevalences were compared with two comparison groups from the literature. Univariable and multivariable analyses were performed.

**Results:**

A total of 249 CCS participated in our study. The OHIP-14 total score had a mean value of 1.94 (sd 4.39), with a median score of 0 (range 0–29). The oral problems ‘oral blisters/aphthae’ (25.9%) and ‘bad odor/halitosis’ (23.3%) were significantly more often reported in CCS than in comparison groups (12% and 12%, respectively). The OHIP-14 score was significantly correlated with the number of self-reported oral health problems (*r* = .333, *p*<0.0005) and dental problems (*r* = .392, *p* <0.0005). In multivariable analysis, CCS with a shorter time since diagnosis (10-19 years vs. ≥30 years) had a 1.47-fold higher risk of ≥1 oral health problem.

**Conclusion:**

Though the perceived oral health is relatively good, oral complications following childhood cancer treatment are prevalent in CCS. This underlines that attention to impaired oral health and awareness on this topic is mandatory and regular visits to the dentist should be a part of long-term follow-up care.

**Supplementary Information:**

The online version contains supplementary material available at 10.1007/s00520-023-07797-1.

## Introduction

Over the past few decades, the overall 5-year survival rate of childhood cancer has increased to approximately 80% [1]. Childhood cancer survivors (CCS) are a growing population, of which 75% experiences one or more late effects arising from their childhood cancer treatment [2]. With the increasing survival rate and high prevalence of late effects, quality of life is gaining more importance as a health outcome in cancer research and treatment.

Among CCS, oral late effects are prevalent [3–5]. Recently, we published two cross-sectional articles on dental developmental disorders, oral health problems, hyposalivation and xerostomia among CCS with a follow-up time of more than 15 years [6, 7]. Of the included CCS, 36.1% experienced at least one dental developmental disorder, 20.4% had increased caries susceptibility, 32.0% had objectively measured hyposalivation and 9.4% experienced xerostomia, the sensation of dry mouth [6, 7]. These long-term effects could affect the oral health-related quality of life (OHRQoL), which represents the quality of life (QoL) in relation to perceived oral health.

While the overall health-related quality of life (HRQoL) of childhood cancer survivors has been measured by several studies [8–10], to our knowledge, only one study examined the OHRQoL [11]. That study reported that childhood cancer survivors aged 8–14 years did not perceive a decreased OHRQoL as compared to their peers [11]. The authors hypothesized that children who have recently completed treatment may report more treatment-related impacts on OHRQoL than children who received treatment a number of years ago [11]. The study had an average follow-up time of 6.2 years [11]. Therefore, the very long-term effects of childhood cancer therapy on OHRQoL are still unclear. To our knowledge, no studies have been published on long-term OHRQoL and self-reported oral complications among CCS of adult age with a follow-up exceeding 15 years. Therefore, the aim of the present study is to determine the prevalence of self-reported oral problems and OHRQoL in CCS at least 15 years after diagnosis and to compare this with the general population.

## Patients and methods

This cross-sectional study is part of the SALI (abbreviation: hypoSALIvation) subproject, part of the multidisciplinary Dutch Childhood Cancer Survivor Study (DCCSS) LATER 2 study [12]. The SALI subproject was approved by the Medical Ethical Committee of the Amsterdam University Medical Center, the Netherlands (protocol number MEC2013_127). Informed consent was obtained from all subjects.

### Participants

In the DCCSS-LATER 2 Study, CCS were included from February 2016 until March 2020 [12]. Survivors were eligible for inclusion if diagnosed with childhood cancer between 1963 and 2001 in one of the seven pediatric oncology centers in the Netherlands before the age of 18 years and have survived at least 5 years since diagnosis of the malignancy. Data collection on childhood cancer diagnosis and treatment information of the underlying cohort of more than 6000 survivors has been described in the LATER 1 methodology paper [13]. In the SALI subproject, participants were included from three of the seven outpatient clinics of DCCSS-LATER 2: Amsterdam University Medical Center (UMC) location VUmc, Leiden University Medical Center (LUMC), and Princess Máxima Center for Pediatric Oncology (PMC). The SALI subproject selected CCS who had received head and neck radiotherapy (H&N RT) or total body irradiation (TBI), and CCS who had not. The inclusion procedure for the SALI subproject has previously been described in detail [7].

### Data collection

Data with regard to gender, age at study, diagnosis [14], age at diagnosis and treatment characteristics have been collected by data managers using a standardized protocol [13]. Treatment exposure data covered treatment for the initial childhood tumor, all recurrences, and any new malignancy. In CCS who received TBI and/or radiotherapy to the head/cranium and/or the neck, radiotherapy was classified as ‘head and neck radiotherapy (H&N RT)’.

To assess self-reported oral health problems and dental problems, survivors were asked to administer the ‘Toegepast-Natuurwetenschappelijk Onderzoek’ (TNO) oral health questionnaire [15], supplemented with three additional questions about the oral hygiene practices of the participants and the number of visits to dental practitioners during the past 12 months. The TNO oral health questionnaire consists of 21 questions about oral health problems (TNO part 1) and dental problems (TNO part 2) during the last 12 months, with the answer options ‘yes’ or ‘no’. We compared our results with data on oral health from control groups of two other studies, that used the same TNO questionnaire [15, 16]. The study by van Gils et al. included 270 acquaintances and partners of celiac disease patients as a comparison group for their study [16]. The study by Kalsbeek et al. investigated the differences in oral health in the general Dutch population (*n*=1309), after the change in the insurance system in 1995 (TNO-cohort) [15]. Furthermore, the CCS in our study administered another questionnaire about the severity of 7 oral problems during the past four weeks, with five-point Likert scales ranging from 0 = never to 4 = very often.

To assess the OHRQoL, participants were asked to administer the Dutch version of the Oral Health Impact Profile-14 (OHIP-14) [17, 18] (Table S1). The OHIP-14 comprises 14 items that measure seven domains of impact of oral health problems on a patient’s life during the past month: functional limitation, physical pain, psychological discomfort, physical disability, psychological disability, social disability and social handicap (Table S1). For each item of the OHIP-14 a five-point Likert scale is used ranging from 0 ‘never’ to 4 ‘very often’ according to the frequency of the impact. The total score can range from 0 (reflecting a high OHRQoL) to 56 (reflecting a low OHRQoL).

### Statistical analysis

All variables were summarized using descriptive statistics. The Mann Whitney U Test was used to examine the associations between gender, H&N RT including TBI, H&N RT without TBI, TBI, and different types of chemotherapy (alkylating agents, vinca alkaloids, anthracyclines, epipodophyllotoxins, platinum compounds, antimetabolites) on one side with self-reported oral problems and OHRQoL on the other side. Spearman’s rank correlation coefficient was used to examine the association between the OHRQoL and self-reported oral problems. A *p* value <0.05 was considered statistically significant. Multivariable Poisson regression analyses with the log-link function and robust standard errors to calculate relative risks were used to evaluate the association between potential risk factors and the occurrence of ≥1 self-reported oral health and dental problem. IBM SPSS version 26.0 (IBM Corp. Armonk, USA) was used to perform data analyses.

## Results

### Patient characteristics

**Fig. 1:**
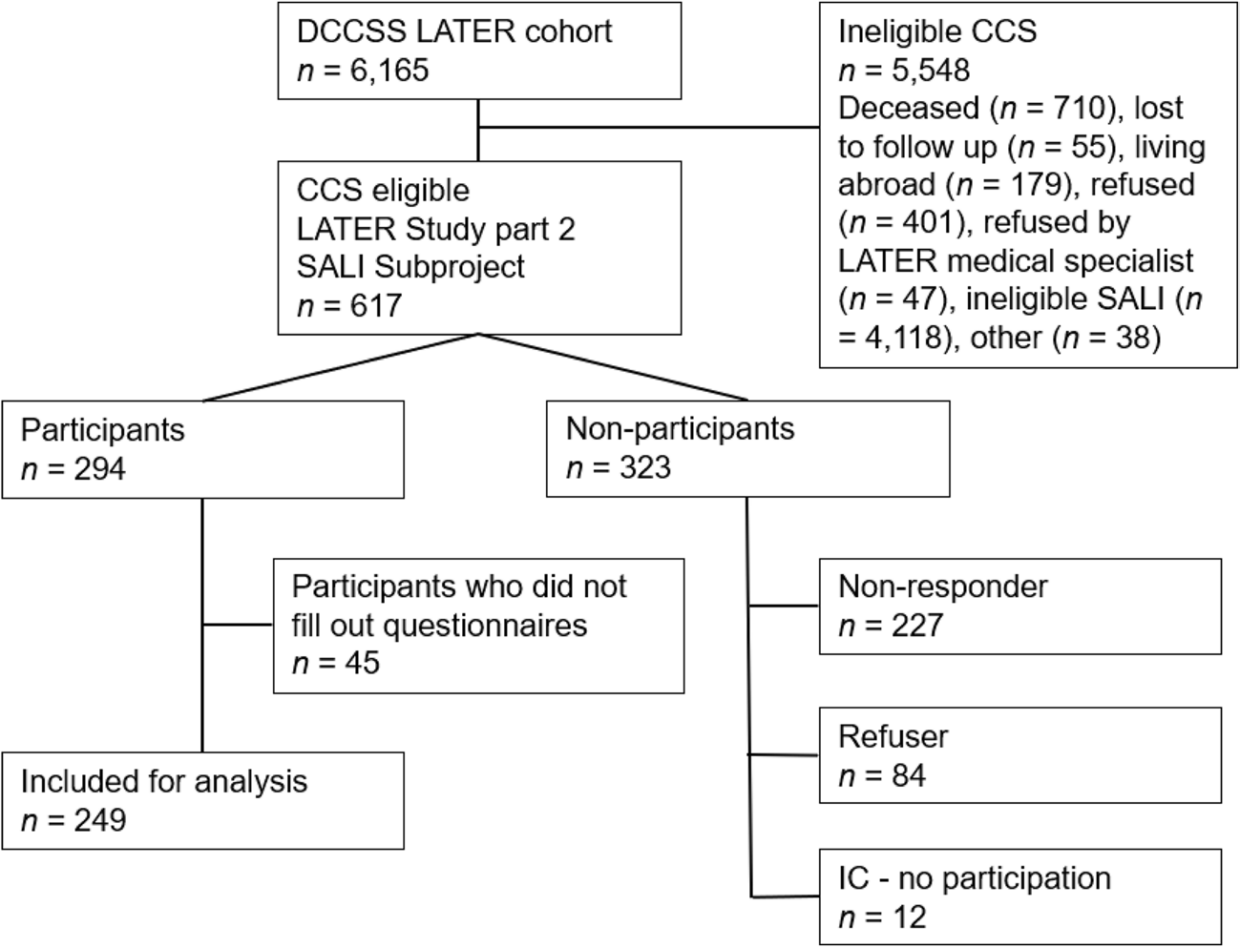
Flowchart of the DCCSS-LATER 2 SALI Study inclusion process. DCCSS LATER; Dutch Childhood Cancer Survivor Study Late Effects Study, IC—no participation; survivors signed informed consent for participation, however, data collection was hampered for different reasons. CCS who were ineligible for the SALI subproject were treated in outpatient clinics of DCCSS LATER 2, which were not participating in the SALI subproject

Figure 1 provides a flowchart of the inclusion process. Of the 617 invited CCS, 249 CCS were included for analysis. Table 1 shows the patient- and treatment-related characteristics of the included CCS. There was an almost equal distribution between men (53.0%) and women (47.0%). A majority of the survivors were diagnosed with a hematological malignancy. The median age at diagnosis was 5.3 years, and the minimum follow-up time between cancer diagnosis and enrollment in the study was 15.9 years with a median time of 25.6 years. The median age at study enrollment was 32.3 years (range 16.8–59.5). Almost all CCS received chemotherapy (96.4%). A smaller proportion received H&N RT (33.5%). The distribution of gender, the type of diagnosis, age at study enrollment, age at diagnosis and time since diagnosis were significantly different between CCS who received H&N RT and CCS who did not receive H&N RT. Table S2 shows the prescribed dose of radiotherapy to different H&N RT fields.

**Table 1:** Patient- and treatment-related characteristics

Patient- and treatment-related characteristics	Total CCS *n* = 249 (100.0) *	H&N RT *n* = 83 (33.5) *	No H&N RT *n* = 165 (66.5) *	*P*
Gender				.007
Male	132 (53.0)	54 (65.1)	77 (46.7)	
Female	117 (47.0)	29 (34.9)	88 (53.3)	
Diagnosis				< .0005
Leukemias, myeloproliferative diseases and myelodysplastic diseases	137 (55.0)	51 (61.4)	86 (52.1)	
Lymphomas and reticuloendothelial neoplasms	43 (17.3)	11 (13.3)	31 (18.8)	
CNS and miscellaneous intracranial and intraspinal neoplasms	16 (6.4)	15 (18.1)	1 (0.6)	
Neuroblastoma and other peripheral nervous cell tumors	3 (1.2)	0 (0.0)	3 (1.8)	
Renal tumors	20 (8.0)	0 (0.0)	20 (12.1)	
Hepatic tumors	1 (0.4)	0 (0.0)	1 (0.6)	
Bone tumors	15 (6.0)	1 (1.2)	14 (8.5)	
Soft tissue and other extraosseous sarcomas	9 (3.6)	3 (3.6)	6 (3.6)	
Germ cell tumors, trophoblastic tumors, and neoplasms of gonads	3 (1.2)	1 (1.2)	2 (1.2)	
Other malignant epithelial neoplasms and malignant melanomas	2 (0.8)	1 (1.2)	1 (0.6)	
Age at study enrollment (y)	32.3 (16.8–59.5)	39.0 (21.1–57.6)	30.2 (16.8–59.5)	
10-17	2 (0.8)	0 (0.0)	2 (1.2)	< .0005
18-29	98 (39.4)	17 (20.5)	80 (48.5)	
30-39	87 (34.9)	29 (34.9)	58 (35.2)	
≥40	62 (24.9)	37 (44.6)	25 (15.2)	
Age at cancer diagnosis (y)	5.3 (0.0–17.0)	7.5 (1.0–16.4)	4.0 (0.0–17.0)	
0-4	118 (47.4)	22 (26.5)	96 (58.2)	< .0005
5-9	77 (30.9)	39 (47.0)	38 (23.0)	
10-14	43 (17.3)	20 (24.1)	22 (13.3)	
≥ 15	11 (4.4)	2 (2.4)	9 (5.5)	
Time since diagnosis (y) ^a^	25.6 (15.9–49.0)	31.5 (16.5–44.7)	24.4 (15.9–49.0)	
0-9	0 (0.0)	0 (0.0)	0 (0.0)	< .0005
10-19	48 (19.3)	10 (12.0)	37 (22.4)	
20-29	120 (48.2)	26 (31.3)	94 (57.0)	
≥ 30	81 (32.5)	47 (56.6)	34 (20.6)	
Type of treatment				
Chemotherapy	240 (96.4)	75 (90.4)	164 (99.4)	.001
Alkylating agents	169 (67.9)	60 (72.3)	108 (65.5)	.315
Vinca alkaloids	204 (81.9)	64 (77.1)	139 (84.2)	.221
Anthracyclines	160 (64.3)	47 (56.6)	112 (67.9)	.093
Epipodophyllotoxins	73 (29.3)	32 (38.6)	40 (24.2)	.026
Platinum compounds	27 (10.8)	9 (10.8)	17 (10.3)	1.000
Antimetabolites	168 (67.5)	62 (74.7)	105 (63.6)	.087
H&N RT ^b, c^	83 (33.5)	83 (100.0)	0 (0.0)	
RT to the head/cranium ^b^	50 (60.2)	50 (60.2)	0 (0.0)	
RT to the neck	7 (8.4)	7 (8.4)	0 (0.0)	
TBI ^b^	27 (32.5)	27 (32.5)	0 (0.0)	
Stem cell transplantation				< .0005
Autologous	10 (4.0)	7 (8.4)	3 (1.8)	
Allogeneic	34 (13.7)	21 (25.3)	12 (7.3)	
SCT unclear	1 (0.4)	0 (0.0)	1 (0.6)	
cGVHD	4 (1.6)	3 (3.6)	1 (0.6)	

Values are presented as n (column %) or median (range). *CCS*, childhood cancer survivors; *CT*, chemotherapy; *RT*, radiotherapy, *SCT*, stem cell transplantation; *cGVHD*, chronic graft-versus-host-disease. Numbers do not always add up to 100% because of rounding. * The numbers of the H&N RT group and the no H&N RT group do not add up to 249 but to 248 because of one of the participants, information about RT is unclear. This participant does not fit in both groups, but is included in the total group. ^a^ At enrollment of the study ^b^ Numbers do not add up to 84 but to 83, because one of the 83 CCS received both RT to the head/cranium and TBI ^c^ 8 of the 83 CCS did not receive CT

Our results were compared with comparison groups from the general population. The comparison group from the study by Van Gils et al. [16] consisted of 270 participants of which 38% were male and 62% were female. The median age was 53 years (range 39–63). The comparison group from the study by Kalsbeek et al. [15] consisted of 1307 participants of which 43% were male and 57% were female. Age categories of 25-34, 34-44 and 44-54 were equally divided with 33% in each group.

### Self-reported outcomes

Table 2 shows the results of the self-reported oral health problems and dental problems of all included CCS, based on the TNO oral health questionnaire. The most frequently reported oral health problems were oral blisters or aphthae (25.9%) and halitosis/bad odor (also known as foetor ex ore) (23.3%). The most frequently reported dental problems were cavities (34.0%), gingival problems (31.6%), and sensitive exposed root surfaces (22.1%). Some problems were significantly more often reported in CCS than in the comparison group of Kalsbeek et al. [15], such as oral blisters/aphthae and bad odor/halitosis. Other problems were more often reported in the comparison group of Kalsbeek et al. [15], such as problems with eating/drinking, missing/loose teeth, sharp teeth, and ‘other dental problems’. The dental problem ‘sensitive exposed root surfaces’ was more often reported in the comparison group of van Gils et al. than in CCS [16]

**Table 2:** Self-reported oral health problems and dental problems in childhood cancer survivors (TNO, part 1 and part 2)

Oral health problems	Total group % (*n*) ^a^	H&N RT % (total *n*) ^b^	No H&N RT % (total *n*) ^b^	*P* *	Van Gils et al. (*n*=270)	*P* *	Kalsbeek et al. (*n*=1309)	*P* *
Problems with eating/drinking	7.6% (19)	9.6% (83)	6.7% (165)	NS	6%	NS	22%	< .0001
Temporomandibular joint complaints	9.0% (22)	8.6% (81)	9.2% (163)	NS	6%	NS	6%	NS
Oral blisters or aphthae	25.9% (64)	15.7% (83)	30.7% (163)	.013	23%	NS	12%	< .0001
Discolorations of the oral mucosa	3.6% (9)	1.2% (83)	4.3% (164)	NS	6%	NS	NR	
Angular cheilitis	11.6% (29)	8.4% (83)	12.7% (165)	NS	11%	NS	NR	
Irritated oral mucosa	3.6% (9)	4.8% (83)	2.4% (164)	NS	7%	NS	NR	
Bad taste	8.9% (22)	6.0% (83)	9.8% (164)	NS	9%	NS	8%	NS
Decreased taste	8.4% (21)	8.4% (83)	7.9% (165)	NS	8%	NS	NR	
Halitosis/bad odor	23.3% (58)	13.3% (83)	27.9% (165)	.010	20%	NS	12%	< .0001
Problems with speaking	4.4% (11)	8.4% (83)	2.4% (165)	.046	4%	NS	NR	
Oral fungus	1.6% (4)	1.2% (83)	1.2% (164)	NS	3%	NS	NR	
Pain	12.1% (30)	8.5% (82)	13.3% (165)	NS	10%	NS	16%	NS
Burning tongue	4.8% (12)	3.6% (83)	5.5% (164)	NS	5%	NS	NR	
Other mouth problems	13.2% (18)	13.5% (52)	13.1% (84)	NS	NR		NR	
Mean number of oral health problems	1.3 ± 1.8	1.1 ± 1.7	1.4 ± 1.7		NR		NR	
Median number of oral health problems	1.0 (0.0–9.0)	0.0 (0.0–8.0)	1.0 (0.0–9.0)	.034 **	NR		NR	
Dental problems	Number % (*n*) ^c^	H&N RT % (total *n*) ^b^	No H&N RT % (total *n*) ^b^	*P* *	Van Gils et al. (*n*=262)	*P* *	Kalsbeek et al. (*n*=1407)	*P* *
Cavities	34.0% (83)	40.5% (79)	30.5% (164)	NS	33%	NS	28%	NS
Gingival problems	31.6% (77)	21.3% (80)	36.2% (163)	.019	29%	NS	27%	NS
Missing/loose teeth	14.8% (36)	12.5% (80)	16.0% (163)	NS	13%	NS	22%	.010
Malposition of teeth	9.4% (23)	8.8% (80)	9.2% (163)	NS	13%	NS	12%	NS
Sharp teeth	8.6% (21)	10.0% (80)	7.3% (164)	NS	12%	NS	15%	.008
Sensitive exposed root surfaces	22.1% (54)	22.8% (79)	22.0% (164)	NS	31%	.026	NR	
Other dental problems	6.1% (7)	9.5% (42)	4.2% (72)	NS	NR		14%	.018
Mean number of dental problems	1.2 ± 1.3	1.2 ± 1.0	1.2 ± 1.3		NR		NR	
Median number of dental problems	1.0 (0.0–5.0)	1.0 (0.0–5.0)	1.0 (0.0–5.0)	.907 **	NR		NR	

Values are presented as % of CCS that answered the question with ‘yes’ (*n* / total *n*) or mean ± SD or median (range). *H&N RT*, head and neck radiotherapy; *NS*, non-significant; *NR*, not reported. ^a^ total *n* varies between 245 and 249, except for ‘Other mouth problems’, total *n*=136. ^b^ *n*= total number of survivors that answered the question ^c^ total *n* varies between 244 and 245, except for ‘Other mouth problems’, total *n*=114. * Chi-2 test. ** Mann Whitney U test

Of the CCS included in our study, 72.3% brushed their teeth at least twice daily and 74.3% used interdental cleaning devices (Table 3). Table 3 also shows the distribution of number of visits to oral health professionals during the past 12 months. Of the CCS, 8% did not visit oral health professionals, whereas 68% visited once or twice a year.

**Table 3:** Self-reported oral hygiene behavior and compliance of childhood cancer survivors

	Total group
Frequency of tooth brushing in times a day (*n*=245)	
﻿ <1	4 (1.6)
﻿ 1	64 (26.1)
﻿ 2	168 (68.6)
﻿ 3	9 (3.7)
Use of interdental cleaning devices (n=245)	
﻿ Toothpicks	121 (49.4)
﻿ Floss	62 (25.3)
﻿ Interdental brushes	57 (23.3)
﻿ Other devices	12 (4.9)
﻿ No devices	63 (25.7)
Use of chewing gum (*n*=249)	36 (14.5)
Use of saliva gel (*n*=249)	1 (0.4)
Distribution of number of visits to oral health professionals during the past 12 months (*n*=243) *	
﻿ 0	20 (8.2)
﻿ 1	62 (25.5)
﻿ 2	100 (41.2)
﻿ 3 to 5	52 (21.4)
﻿ 6 to 9	9 (3.7)

Values are presented as n (column %). * Visits to dentist, oral hygienist or oral and maxillofacial surgeon including control visits

**Fig. 2:**
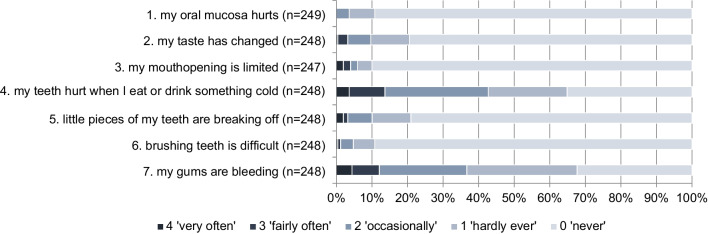
Distribution of responses of childhood cancer survivors to different items on the frequency of self-reported oral problems on a 5-point Likert Scale ranging from 0 (never) to 4 (very often)

Figure 2 shows the distribution of responses to different items of the questionnaire with seven self-reported oral problems. The most frequently reported oral health problems were painful teeth when exposed to cold stimuli in food or drinks (3.6% ‘very often’ and 10.1% ‘often’) and gingival bleeding (4.4% ‘very often’ and 7.7% ‘often’).

**Fig. 3:**
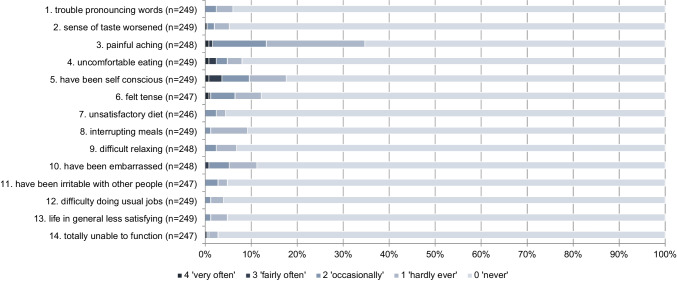
Distribution of responses of childhood cancer survivors to the 14 different items of the Oral Health Impact Profile-14

Figure 3 shows the distribution of responses to the different items of the OHIP-14. The domains physical pain (painful aching and uncomfortable eating), psychological discomfort (having been self-conscious and felt tense) and psychological disability (having difficulty relaxing and having been embarrassed) were most frequently reported as being impacted. The OHIP-14 total score had a mean value of 1.94 (sd 4.39), with a median score of 0 (range 0–29) (*n*=237).

### Association between patient- and treatment-related factors and OHRQoL, oral health problems and dental problems

Table 4 shows the univariable analyses between patient- and treatment-related factors and OHRQoL, oral health problems, and dental problems. Time since diagnosis (*r* = − .148, *p* = .019) and age at study enrollment (*r* = −.151, *p* = .017) were significantly negatively associated with the number of oral health problems, reflecting less oral health problems in CCS with a longer time since diagnosis and older age. Survivors who received H&N RT, including TBI, had a significantly lower number of oral health problems (median: 0) than survivors who did not receive H&N RT (median: 1). The type of chemotherapy, H&N RT without TBI, and TBI were not significantly associated with OHRQoL, number of oral health problems and number of dental problems (Table S3). The OHIP-14 score was significantly correlated with the number of oral health problems (*r* = .333, *p*<0.0005) and with the number of dental problems (*r* = .392, *p* <0.0005), reflecting that survivors with more oral health and dental problems more often had a decreased OHRQoL (Table 4).

**Table 4:** Associations between patient- and treatment-related factors and OHRQoL, oral health problems and dental problems in childhood cancer survivors

	OHRQoL	Number of oral health problems	Number of dental problems
Age at diagnosis, continuous ^a^	*r* = −.006 (*p* = .928)	*r* = - .075 (*p* = .237)	*R* = .029 (*p* = .650)
Time since diagnosis, continuous ^a^	*r* = − .050 (*p* = .441)	*r* = - .148 (*p* = .019)	*R* = .086 (*p* = .175)
Age at study enrollment, continuous ^a^	*r* = − .043 (*p* = .513)	*r* = – .151 (*p* = .017)	*R* = .096 (*p* = .131)
Gender ^b^	*p* = .755	*p* = .316	*P* = .976
Male ^c^	2.0 (4.6) | 0.0 (0–29)	1.2 (1.7) | 1.0 (0–9)	1.2 (1.3) | 1.0 (0–5)
Female ^c^	1.8 (4.2) | 0.0 (0–29)	1.4 (1.8) | 1.0 (0–9)	1.2 (1.3) | 1.0 (0–5)
H&N RT incl. TBI ^b^	*p* = .339	*p* = .034	P = .907
Yes ^c^	2.7 (5.9) | 0.0 (0–29)	1.1 (1.7) | 0 (0–8)	1.2 (1.2) | 1.0 (0–5)
No ^c^	1.5 (3.3) | 0.0 (0–22)	1.4 (1.7) | 1.0 (0–9)	1.2 (1.3) | 1.0 (0–5)
Number of oral health problems ^a^	*r* = .333, *p* < .0005		
Number of dental problems ^a^	*r* = .392, *p* < .0005		

^a^ Spearman’s rank correlation coefficient, ^b^ Mann Whitney U Test, ^c^ values are presented as mean (*SD*) | median (range)

We performed Poisson regression analysis to evaluate the possible role of patient- and treatment-related characteristics in the prevalence of ≥1 oral health problem and ≥1 dental problem (Table 5). The relative risk of ≥1 oral health problem was associated with time since diagnosis (RR ≥30 years vs. 10-19 years, .678; 95% CI, .491 to .938). In contrast to the result in univariable analysis, H&N RT incl. TBI was not significantly associated with ≥1 oral health problem (RR H&N RT incl. TBI vs. no H&N RT incl. TBI, .859; 95% CI, .653 to 1.129). Gender and age at diagnosis were not significantly associated with ≥1 oral health problem either. The relative risk of ≥1 dental problem was not significantly associated with gender, age at diagnosis, time since diagnosis and H&N RT incl. TBI.

**Table 5:** Poisson regression analysis for the number of oral health and dental problems, measured by TNO oral health questionnaire in childhood cancer survivors (*n* = 248)

Variable	Number of survivors ^a^	≥1 oral health problem (*n*)	Risk ratio	95% CI	*p*	≥1 dental problem (*n*)	Risk Ratio	95% CI	*p*
Gender									
Male	131	69	(ref)			82	(ref)		
Female	117	70	1.124	.897 – 1.408	.312	69	.943	.764 – 1.165	.586
Age at diagnosis (y)									
0-4	118	71	(ref)			70	(ref)		
5-9	77	39	.910	.687 – 1.205	.509	46	.975	.753 – 1.262	.847
10-17	53	29	.915	.687 – 1.220	.545	35	1.073	.830 – 1.387	.591
Time since diagnosis (y)									
10-19	47	33	(ref)			24	(ref)		
20-29	120	70	.863	.675 – 1.102	.237	74	1.267	.916 – 1.752	.152
≥30	81	36	.678	.491 – .938	.019	53	1.340	.952 – 1.887	.093
H&N RT incl TBI									
No	165	99	(ref)			98	(ref)		
Yes	83	39	.859	.653 – 1.129	.275	52	1.000	.781 – 1.280	.999

(ref); reference category; *H&N RT*, head and neck radiotherapy; *TBI*, total body irradiation

## Discussion

The present study describes self-reported oral complications and OHRQoL in CCS with a long time since diagnosis (>15 years). The OHRQoL is significantly associated with the number of self-reported oral health problems and the number of dental problems, but the OHIP-14 score was low, implicating that perceived OHRQoL overall was relatively good. Childhood cancer treatment-related factors such as different types of chemotherapy or treatment with H&N RT were not significantly associated with a decreased OHRQoL or a higher number of oral health problems and dental problems. In multivariable analysis, CCS with a shorter time since diagnosis (10-19 years vs. ≥30 years) had a 1.47-fold higher risk of ≥1 oral health problem.

Overall, the prevalence of oral health problems and dental problems in CCS was not significantly different from the comparison groups from the general population. Only oral health problems such as oral blisters/aphthae and bad odor/halitosis were more often reported in CCS than in the comparison groups. However, the comparison of oral and dental problems in CCS with the general population is hampered by the fact that for several problems the prevalences in the two comparison groups differ considerably (Table 2). Also, in the study by Kalsbeek et al., not all oral health problems assessed in our CCS group were studied. Moreover, the comparison groups from the general population have a higher age, which may account for the higher prevalence of oral health and dental problems.

H&N RT is associated with an increased risk of oral health problems in some studies [3–5]. Surprisingly, we found that H&N RT was associated with a lower number of oral health problems in univariable analysis. However, CCS who received H&N RT were significantly older at study enrollment and had a longer follow-up time as compared to CCS who did not receive H&N RT, two factors also associated with decreased risk of oral health problems in univariable analysis. Indeed, in multivariable analysis adjusted for follow-up time and also for age at diagnosis and gender, H&N RT was no longer significantly associated with any oral health problem. Our hypothesis for a decreased risk of ≥1 oral health problem in CCS with longer follow-up time is that survivors, due to their treatment at a young age, might have an increased pain threshold and/or have learned to cope with oral health complications after cancer treatment. Older survivors, or survivors with a longer follow-up time, who report less oral health and dental problems, may have gotten used to their impaired oral health. Another explanation could be that certain oral and dental problems have been addressed by dentists over time and are therefore no longer experienced by CCS.

The results of the present study are based on self-reported oral and dental problems. However, self-reported problems are not identical to oral health parameters, objectively determined by an oral health professional. In one of our recently published LATER-2 articles, we evaluated the oral health of CCS based on data provided by their dentists [6]. 154 Dentists of CCS participated, representing 61.8% of the study group of the present article. Overall, oral problems were less frequently reported by the dentists of CCS when compared to subjective oral complications as reported by CCS in the present study. In 1.8% of CCS, dentists reported that complaints of altered taste were present [6], whereas 8.9% of CCS reported experiencing a bad taste and 8.4% reported experiencing a decreased taste sensation. In 4.5% of CCS, dentists reported that temporomandibular dysfunction (TMD) was present [6], whereas 9% of CCS reported experiencing temporomandibular joint complaints. This means that dentists not always seem to be aware of all patients’ subjective complaints. For the detection of dry mouth, we saw a similar trend. In 4.0% of CCS, dentists reported that complaints of xerostomia were present [6], while 9.4% of CCS experienced xerostomia [7].

OHRQoL represents the quality of life (QoL) in relation to perceived oral health, which is part of the general health-related quality of life (HRQoL). Although general HRQoL numerous measures exist [19], they do not address OHRQoL properly, and therefore OHRQoL has to be measured with a separate, specialized questionnaire [17, 18]. In a study among Dutch adult CCS, it was reported that both male and female CCS had worse HRQoL than the general population [8]. That study did not focus on oral symptoms. Significant risk factors for impaired physical HRQoL were female sex, older age at diagnosis, not having a partner, low educational attainment, disease recurrence and exposure to radiotherapy, especially irradiation of the lower extremity [8]. In the present study, no significant associations were found between patient- and treatment-related factors and the OHRQoL. However, we cannot exclude that possible associations between OHRQoL and sociodemographic factors do exist, as these factors were not assessed in the present study.

Some studies evaluated OHRQoL among childhood cancer survivors or other pediatric disease populations and associated factors. Dental discoloration, untreated caries lesions and oral mucositis negatively impacted the OHRQoL [20, 21]. However, in a study among children having survived childhood cancer, the type of cancer and its treatment were not associated with a decreased OHRQoL compared to children without a cancer history [11]. A recent review [22] on OHRQoL in children and adult patients with a hematological malignancy reported that functional limitations because of problems with oral mucosal tissues, the dentition, or dentures, seem to have a larger negative impact on the OHRQoL than social aspects associated with oral health problems. Similar to that review, in the present study, the questions in the domain of physical pain were given the highest scores (Figure 3, Table S1).

In survivors of head and neck cancer (HNC), quality of life (QoL) and OHRQoL are affected by oral complications resulting from the therapy. Compared to the present study, in a study among 90 adult HNC survivors (age at diagnosis and treatment >18 years and mean follow-up time of 35.59 ± 37.60 months), the OHIP-14 score was much higher, 23.98 ± 12.55, implicating poorer OHRQoL [23]. Hyposalivation and advanced-stage tumors were associated with greater severity of the negative impact, and a longer time since oncological treatment was associated with a lower OHIP-14 score, implicating better OHRQoL [23]. In a study among 216 adult HNC survivors (59.7% had a follow-up time between 0 and 10 years), moderately poor self-rated dental health and general health were significantly associated with poorer OHRQoL, which is in line with the present study [24]. HNC survivors also had a higher risk of reporting oral functional problems compared to the general population [24]. We hypothesize that the less impaired OHRQoL in CCS after HNRT may be explained by the long follow-up time and the fact that they may have gotten used to these problems present since childhood. The significant association between a longer time since diagnosis and a lower number of self-reported oral health problems supports this hypothesis. Another explanation for worse OHRQoL in HNC patients is the often a high dose of H&N RT prescribed (50–70 Gray (Gy)) leading to serious, life-long complications [25, 26]. In the present study, CCS received a lower prescribed mean dose of 36 Gy to the head/cranium and 32 Gy to the neck. The lower dose may have allowed CCS to (partly) recover from complications following radiotherapy and have a better OHRQoL. Also, the oral cavity may not has been included in all fields of H&N RT applied in our study group.

Although CCS are at increased risk for treatment-related oral complications, the frequency of visits of CCS to the dental practice in Minneapolis was below recommended levels [27]. Within the last year, 60.4% of survivors reported a dental visit [27]. This is confirmed by a study from Australia, that reported that one-third of allogeneic HSCT survivors are not receiving regular dental reviews in accordance with HSCT long-term follow-up guidelines [28]. In the present study, 92% of the CCS reported having visited an oral care provider at least once during the past 12 months, which could possibly explain the relatively good OHRQoL. However, a poorer OHRQoL was associated with the number of oral health and dental problems, that were reported in up to 34% of CCS. In general, regular visits to the dental practice and awareness among dentists of prior childhood cancer and subsequent prevention and management of oral complications in CCS may improve the OHRQoL.

### Strengths and limitations

The SALI Subproject selected CCS who had received H&N RT and CCS who had not. Therefore the present study should be interpreted with caution, as this cohort is not representative of the total Dutch CCS cohort. Selection bias could exist, as CCS experiencing more oral and dental problems may have been more likely to participate in the present study. A strength of the present study is that for collecting data on self-reported parameters, validated questionnaires were used. We were able to explore the possible relation between self-reported oral health problems and dental problems with OHRQoL and to relate this to the objective data on childhood cancer treatment. Detailed and complete data on the treatment characteristics such as type of diagnosis and type of treatment were collected. In addition, the cohort had a long follow-up time of more than 15 years. In the present study, we were not able to include a sex- and age-matched control group. However, to compare the outcomes with the general population, we used data of two studies that used the same questionnaires to assess self-reported outcomes on oral health and dental problems. Future studies on the self-reported oral and dental problems of CCS should include a control group, preferably healthy siblings of the CCS.

## Conclusion

Among CCS in the present study, OHRQoL was perceived as relatively good. OHRQoL was negatively affected by a higher number of oral health and dental problems. Oral blisters/aphthae and halitosis were more prevalent in CCS than in the general population. A younger age at study enrollment and a shorter time since childhood cancer diagnosis were associated with a higher number of oral health problems. Though the perceived oral health is relatively good, it has been observed that oral complications following childhood cancer treatment in CCS are prevalent. This underlines that paying attention to impaired oral health is important and regular visits to the dentist should be a part of long-term follow-up care. Education on the long-term effects of childhood cancer therapy on oral health and awareness of this topic among pediatric oncologists, dental practitioners and survivors themselves is important to improve oral health in CCS.

## Supplementary information

ESM 1: **Table S1**. 14 items of the Oral Health Impact Profile, OHIP-14. **Table S2.** Prescribed dose of radiotherapy to different fields of the head and neck (in Gray). **Table S3.** Associations between treatment-related factors and OHRQoL, oral health problems and dental problems in childhood cancer survivors.

## Data Availability

The data that support the findings of this study will be stored for at least 10 years. Request for data can be made via TDC LATER, with an application of intent (M.vanderHeiden@prinsesmaximacentrum.nl).

## Code availability

Does not apply.

## References

[1] O'Leary M, Krailo M, Anderson JR, Reaman GH (2008). Progress in childhood cancer: 50 years of research. Semin Oncol.

[2] Geenen MM, Cardous-Ubbink MC, Kremer LCM (2007). Medical assessment of adverse health outcomes in long-term survivors of childhood cancer. J Am Med Assoc.

[3] Kaste SC, Goodman P, Leisenring W, Stovall M, Hayashi RJ, Yeazel M (2009). Impact of radiation and chemotherapy on risk of dental abnormalities: a report from the childhood cancer survivor study. Cancer Interdiscip Int J Am Cancer Soc.

[4] Milgrom SA, van Luijk P, Pino R (2021). Salivary and dental complications in childhood cancer survivors treated with radiation therapy to the head and neck: a Pediatric Normal Tissue Effects in the Clinic (PENTEC) Comprehensive Review. Int J Radiat Oncol Biol Phys.

[5] Seremidi K, Kloukos D, Polychronopoulou A (2019). Late effects of chemo and radiation treatment on dental structures of childhood cancer survivors. A systematic review and meta-analysis. Head Neck.

[6] Stolze J, Vlaanderen KCE, Holtbach FCED (2021). Long-term effects of childhood cancer treatment on dentition and oral health: a dentist survey study from the DCCSS LATER 2 Study. Cancers (Basel).

[7] Stolze J, Teepen JC, Raber-Durlacher JE (2022). Prevalence and risk factors for hyposalivation and xerostomia in childhood cancer survivors following different treatment modalities-a Dutch Childhood Cancer Survivor Study Late Effects 2 Clinical Study (DCCSS LATER 2). Cancers (Basel).

[8] van Erp LME, Maurice-Stam H, Kremer LCM (2021). Health-related quality of life in Dutch adult survivors of childhood cancer: a nation-wide cohort study. Eur J Cancer.

[9] Zeltzer LK, Lu Q, Leisenring W (2008). Psychosocial outcomes and health-related quality of life in adult childhood cancer survivors: a report from the childhood cancer survivor study. Cancer Epidemiol biomarkers Prev a Publ Am Assoc Cancer Res cosponsored by Am Soc Prev Oncol.

[10] Rueegg CS, Gianinazzi ME, Rischewski J (2013). Health-related quality of life in survivors of childhood cancer: the role of chronic health problems. J Cancer Surviv.

[11] Wogelius P, Rosthøj S, Dahllöf G, Poulsen S (2011). Oral health-related quality of life among survivors of childhood cancer. Int J Paediatr Dent.

[12] Feijen EAM, Teepen JC, van Dulmen-den Broeder E, et al (2023) Clinical evaluation of late outcomes in Dutch childhood cancer survivors: Methodology of the DCCSS LATER 2 study. Pediatr Blood Cancer e30212. 10.1002/pbc.30212 10.1002/pbc.30212 36651687

[13] Teepen JC, Kok JL, Feijen EAM (2022). Questionnaire- and linkage-based outcomes in Dutch childhood cancer survivors: Methodology of the DCCSS LATER study part 1. Cancer Med..

[14] Steliarova-Foucher E, Stiller C, Lacour B, Kaatsch P (2005). International classification of childhood cancer, third edition. Cancer.

[15] Kalsbeek H, Poorterman J, Kivit M (2003). Tandheelkundige verzorging volwassen ziekenfondsverzekerden.

[16] van Gils T, Bouma G, Bontkes HJ (2017). Self-reported oral health and xerostomia in adult patients with celiac disease versus a comparison group. Oral Surg Oral Med Oral Pathol Oral Radiol.

[17] Slade GD, Spencer AJ (1994). Development and evaluation of the Oral Health Impact Profile. Community Dent Health.

[18] Van Der Meulen MJ, John MT, Naeije M, Lobbezoo F (2008). The Dutch version of the Oral Health Impact Profile (OHIP-NL): translation, reliability and construct validity. BMC Oral Health.

[19] McDougall J, Tsonis M (2009). Quality of life in survivors of childhood cancer: a systematic review of the literature (2001-2008). Support Care Cancer.

[20] Vidigal EA, Abanto J, Haddad AE (2020). Oral health-related quality of life among pediatric liver transplant candidates. Braz Oral Res.

[21] Barkokebas A, Silva IHM, de Andrade SC (2015). Impact of oral mucositis on oral-health-related quality of life of patients diagnosed with cancer. J oral Pathol Med Off Publ Int Assoc Oral Pathol Am Acad Oral Pathol.

[22] Stolze J, Vlaanderen KCE, Raber-Durlacher JE, Brand HS (2020). The impact of hematological malignancies and their treatment on oral health-related quality of life as assessed by the OHIP-14: a systematic review. Odontology.

[23] Soldera EB, Ortigara GB, Bonzanini LIL (2020). Clinical and sociodemographic factors associated with oral health-related quality of life in survivors of head and neck cancer. Head Neck.

[24] Andreassen R, Jönsson B, Hadler-Olsen E (2022). Oral health related quality of life in long-term survivors of head and neck cancer compared to a general population from the seventh Tromsø study. BMC Oral Health.

[25] Epstein JB, Thariat J, Bensadoun R-J (2012). Oral complications of cancer and cancer therapy. CA Cancer J Clin.

[26] Vissink A, Jansma J, Spijkervet FKL (2003). Oral sequelae of head and neck radiotherapy. Crit Rev Oral Biol Med.

[27] Yeazel MW, Gurney JG, Oeffinger KC (2004). An examination of the dental utilization practices of adult survivors of childhood cancer: a report from the Childhood Cancer Survivor Study. J Public Health Dent.

[28] Dyer G, Brice L, Schifter M (2018). Oral health and dental morbidity in long-term allogeneic blood and marrow transplant survivors in Australia. Aust Dent J.

