# Supplementary material for: Self-reported outcomes on oral health and oral health-related quality of life in long-term childhood cancer survivors—A DCCSS-LATER 2 Study
Source: Support Care Cancer. 2023 May 19;31(6):344. doi: 10.1007/s00520-023-07797-1 (PMC10198911; doi:10.1007/s00520-023-07797-1)
Supplement: Supplementary file 1 — Table S1. 14 items of the Oral Health Impact Profile, OHIP-14. Table S2. Prescribed dose of radiotherapy to different fields of the head and neck (in Gray). Table S3. Associations between treatment-related factors and OHRQoL, oral health problems and dental problems in childhood cancer survivors. [file 520_2023_7797_MOESM1_ESM.docx]

Supplementary Results

**Table S1**. 14 items of the Oral Health Impact Profile, OHIP-14.

| Functional limitation | Have you had trouble pronouncing any words because of problems with your teeth, mouth or dentures? |
| --- | --- |
|  | Have you felt that your sense of taste has worsened because of problems with your teeth, mouth or dentures? |
| Physical pain | Have you had painful aching in your mouth? |
|  | Have you found it uncomfortable to eat any foods because of problems with your teeth, mouth or dentures? |
| Psychological discomfort | Have you been self-conscious because of your teeth, mouth or dentures? |
|  | Have you felt tense because of problems with your teeth, mouth or dentures? |
| Physical disability | Has your diet been unsatisfactory because of problems with your teeth, mouth or dentures? |
|  | Have you had to interrupt meals because of problems with your teeth, mouth or dentures? |
| Psychological disability | Have you found it difficult to relax because of problems with your teeth, mouth or dentures? |
|  | Have you been a bit embarrassed because of problems with your teeth, mouth or dentures? |
| Social disability | Have you been a bit irritable with other people because of problems with your teeth, mouth or dentures? |
|  | Have you had difficulty doing your usual jobs because of problems with your teeth, mouth or dentures? |
| Social handicap | Have you felt that life in general was less satisfying because of problems with your teeth, mouth or dentures? |
|  | Have you been totally unable to function because of problems with your teeth, mouth or dentures? |

**Table S2.** Prescribed dose of radiotherapy to different fields of the head and neck (in Gray).

|  | Number (%) | Mean (sd) | Median (range) ^b^ |
| --- | --- | --- | --- |
| H&N RT | 83 (100.0) | 27.74 (18.55) | 25.00 (5.00-100.80) |
| RT to the head/cranium ^a^ | 50 (60.2) | 36.54 (17.19) | 25.00 (18.00-100.80) |
| RT to the neck | 7 (8.4) | 32.74 (9.48) | 39.40 (19.80-40.00) |
| TBI ^a^ | 27 (32.5) | 9.13 (2.30) | 7.50 (5.00-12.00) |

^a^ Numbers do not add up to 84 but to 83, because one of the 83 CCS received both RT to the head/cranium and TBI  ^b^ Only one of the participants received a dose of 100.80 Gy. The second highest dose was 64.80 Gy.

**Table S3.** Associations between treatment-related factors and OHRQoL, oral health problems and dental problems in childhood cancer survivors.

|  | OHRQoL (OHIP total score) | Number of oral health problems | Number of dental problems |
| --- | --- | --- | --- |
| H&N RT without TBI ^a^ | p = .755 | p = .095 | p = .758 |
| Yes ^b^ | 2.7 (5.9) \| 0.0 (0-29) | 1.1 (1.8) \| 0.0 (0-8) | 1.3 (1.3) \| 1.0 (0-5) |
| No ^b^ | 1.7 (3.8) \| 0.0 (0-29) | 1.4 (1.8) \| 1.0 (0-9) | 1.2 (1.3) \| 1.0 (0-5) |
| TBI ^a^ | p = .328 | p = .881 | p = .438 |
| Yes ^b^ | 2.6 (6.0) \| 0.0 (0-29) | 1.0 (1.6) \| 0.0 (0-7) | 0.9 (0.9) \| 1.0 (0-3) |
| No ^b^ | 2.1 (5.1) \| 0.0 (0-29) | 1.0 (1.6) \| 0.0 (0-8) | 1.2 (1.3) \| 1.0 (0-5) |
| Alkylating agents ^a^ | p = .177 | p = .947 | p = .802 |
| Yes ^b^ | 2.1 (4.8) \| 0.0 (0-29) | 1.4 (1.9) \| 1.0 (0-9) | 1.2 (1.3) \| 1.0 (0-5) |
| No ^b^ | 1.5 (3.3) \| 0.0 (0-18) | 1.2 (1.4) \| 1.0 (0-5) | 1.2 (1.3) \| 1.0 (0-5) |
| Vinca alkaloids ^a^ | p = .700 | p = .120 | p = .860 |
| Yes ^b^ | 1.8 (4.0) \| 0.0 (0-29) | 1.4 (1.8) \| 1.0 (0-9) | 1.2 (1.3) \| 1.0 (0-5) |
| No ^b^ | 2.5 (5.9) \| 0.0 (0-29) | 1.0 (1.4) \| 0.0 (0-5) | 1.2 (1.4) \| 1.0 (0-5) |
| Anthracyclines ^a^ | p = .682 | p = .424 | p = .494 |
| Yes ^b^ | 1.7 (4.0) \| 0.0 (0-29) | 1.3 (1.8) \| 1.0 (0-9) | 1.2 (1.2) \| 1.0 (0-5) |
| No ^b^ | 2.3 (5.0) \| 0.0 (0-29) | 1.4 (1.7) \| 1.0 (0-8) | 1.3 (1.4) \| 1.0 (0-5) |
| Epipodophyllotoxins ^a^ | p = .179 | p = .209 | p = .412 |
| Yes ^b^ | 2.2 (3.9) \| 0.0 (0-18) | 1.5 (2.0) \| 1.0 (0-9) | 1.3 (1.3) \| 1.0 (0-5) |
| No ^b^ | 1.8 (4.6) \| 0.0 (0-29) | 1.2 (1.7) \| 1.0 (0-9) | 1.2 (1.3) \| 1.0 (0-5) |
| Platinum Compounds ^a^ | p = .083 | p = .269 | p = .432 |
| Yes ^b^ | 3.1 (5.5) \| 1.0 (0-24) | 2.0 (2.7) \| 1.0 (0-9) | 1.5 (1.6) \| 1.0 (0-5) |
| No ^b^ | 1.8 (4.2) \| 0.0 (0-29) | 1.2 (1.6) \| 1.0 (0-9) | 1.2 (1.3) \| 1.0 (0-5) |
| Antimetabolites ^a^ | p = .989 | p = .600 | p = .665 |
| Yes ^b^ | 2.1 (4.8) \| 0.0 (0-29) | 1.3 (1.9) \| 1.0 (0-9) | 1.2 (1.3) \| 1.0 (0-5) |
| No ^b^ | 1.7 (3.5) \| 0.0 (0-22) | 1.3 (1.6) \| 1.0 (0-8) | 1.3 (1.4) \| 1.0 (0-5) |

*^a^ Mann-Whitney U Test, ^b^ values are presented as mean (sd) | median (range).*
